# Electron paramagnetic resonance measurements of absorbed dose in teeth from citizens of Ozyorsk

**DOI:** 10.1007/s00411-014-0527-8

**Published:** 2014-03-07

**Authors:** A. Wieser, E. Vasilenko, E. Aladova, P. Fattibene, N. Semiochkina, M. Smetanin

**Affiliations:** 1 Institute of Radiation Protection, Helmholtz Zentrum München - German Research Center for Environmental Health, 85764 Neuherberg, Germany; 2 Southern Urals Biophysics Institute, 456780 Ozyorsk, Russia; 3 Istituto Superiore di Sanità, Viale Regina Elena 299, 00162 Rome, Italy

**Keywords:** Dose reconstruction, Mayak Production Association, Background radiation, Radioactive noble gas isotopes, EPR, Tooth enamel

## Abstract

In 1945, within the frame of the Uranium Project for the production of nuclear weapons, the Mayak nuclear facilities were constructed at the Lake Irtyash in the Southern Urals, Russia. The nuclear workers of the Mayak Production Association (MPA), who lived in the city of Ozyorsk, are the focus of epidemiological studies for the assessment of health risks due to protracted exposure to ionising radiation. Electron paramagnetic resonance measurements of absorbed dose in tooth enamel have already been used in the past, in an effort to validate occupational external doses that were evaluated in the Mayak Worker Dosimetry System. In the present study, 229 teeth of Ozyorsk citizens not employed at MPA were investigated for the assessment of external background exposure in Ozyorsk. The annually absorbed dose in tooth enamel from natural background radiation was estimated to be (0.7 ± 0.3) mGy. For citizens living in Ozyorsk during the time of routine noble gas releases of the MPA, which peaked in 1953, the average excess absorbed dose in enamel above natural background was (36 ± 29) mGy, which is consistent with the gamma dose obtained by model calculations. In addition, there were indications of possible accidental gaseous MPA releases that affected the population of Ozyorsk, during the early and late MPA operation periods, before 1951 and after 1960.

## Introduction

In 1945, the former Soviet Union initiated the so-called Uranium Project for the production of nuclear weapons. More specifically, in November of that year, construction of the first of five industrial nuclear reactors for weapons-grade plutonium production started and the city of Ozyorsk was the residence of the workers. The start-up of the first reactor was in June 1948, and the first weapons-grade plutonium was extracted in February 1949. The last of the five reactors started to operate in September 1952. All the nuclear facilities were operated by the Mayak Production Association (MPA). Recently, the nuclear workers (Shilnikova et al. 2003; Azizova et al. 2010) and the populations affected by liquid (Schonfeld et al. 2013) and gaseous (Koshurnikova et al. 2002) radioactive releases of the MPA nuclear facilities have been the focus of epidemiological studies, for the assessment of health risks due to protracted exposure to ionising radiation. The health risks of the MPA workers due to external occupational exposures are assessed by means of specific organ doses that are based on occupational dose monitoring with film badges and thermoluminescence dosimeters (TLD). In the Mayak Worker Dosimetry Study, exposure scenarios were defined for the workplaces of approximately 19,000 MPA workers, in an effort to calculate dose conversion factors for the reconstruction of organ doses from the archive records of occupational dosimetry (Vasilenko et al. 2007). Electron paramagnetic resonance (EPR) measurements of absorbed dose in tooth enamel of these workers were used to validate and improve the defined exposure scenarios (Wieser et al. 2006). For this, the occupational dose of a worker was assessed by measuring the absorbed dose in tooth enamel, and subtracting individual background dose and any contribution to dose from medical exposure (Wieser et al. 2011).

In the present study, the background dose in teeth of citizens of Ozyorsk who were never employed by MPA was investigated. The city of Ozyorsk is located at a distance of about 15 km north-west to the MPA nuclear complex. According to the first census in 1959, about 50,000 citizens were living in Ozyorsk with approximately half of the adults working at the MPA. Beside exposure due to natural (background) and medical sources of ionising radiation, the teeth of citizens not employed by MPA could have been also exposed by routine and accidental radioactive MPA releases to the atmosphere. Regular measurements of environmental contamination as well as of liquid and atmospheric releases started after 1958, and practically all information on earlier significant releases, resulted from model calculations performed by MPA experts on the basis of the data on the radioactive inventory and operation modes of the nuclear facilities.

From 1949 to 1956, liquid radioactive waste was released from the MPA radiochemical plant into the Techa River, with an estimated activity of 114 PBq discharged mainly in 1950–1951, including 52 PBq accidentally released from September to November 1951 (Glagolenko et al. 2008a; Degteva et al. 2012). The liquid radioactive waste consisted of about 3 % of the long-lived radionuclides ^90^Sr and ^137^Cs. Once incorporated, ^90^Sr was integrated in tooth enamel (Shishkina et al. 2013) where it resulted in an absorbed dose to the enamel of up to 20 Gy, for those residents who lived along the Techa River and whose tooth calcification began in 1951, the year of maximal release (Tolstykh et al. 2003).

It has been estimated that between 1948 when the first MPA reactor was put into operation and 1990, about 179,000 PBq of noble gas radionuclides were released routinely into the atmosphere (Glagolenko et al. 2008b). Most releases (90 %) occurred in 1951–1956 with a maximal annual discharge of 37,000 PBq in 1953. About 50 % of those releases consisted of the short-lived gaseous radionuclide ^41^Ar, which in turn was responsible for about 90 % of the external gamma dose from these releases (Glagolenko et al. 2008c), depending on the distance and direction from the site of atmospheric releases. At the MPA central physical laboratory in the West, and the Meteostation in the north-east part of the city of Ozyorsk, located north-west of the site of releases, cumulated outdoor external doses of 13 and 19 mGy were estimated, respectively. In contrast, about 10 km north-east from the site of releases in the village of Metlino, estimated cumulated outdoor external dose was 132 mGy, while at the MPA production site itself it was 708 mGy. Between 1948 and 1964, seven accidents occurred at the first MPA reactor with overheating and melting of the uranium core and subsequent shutdown of the reactor. Eight additional accidents occurred at the four other reactors between 1955 and 1975 (Glagolenko et al. 2008b). These accidents resulted in atmospheric releases of a significant amount of radioactive aerosols and gaseous fission products (radioisotopes of noble gases and radioactive iodine). Estimates of the discharged activities are currently not available. Note that if radioactive equilibrium is assumed in a reactor, it can be expected that about 90 % of the radioisotopes of noble gases released were ^133^Xe.

Additionally, a large accident with the release of mixed fission products occurred in September 1957, an accident known as the Kyshtym accident (Peremyslova et al. 2004). After explosion of a storage tank for liquid radioactive waste, due to failure of the cooling system, about 740 PBq of activity was released into the atmosphere, mainly the short-lived beta-emitters ^144^Ce and ^106^Ru, the gamma-emitter ^95^Zr, and the longer living beta-emitter ^90^Sr. These releases created the so-called East Urals Radioactive Trace (EURT), a contaminated area extending to about 300 km to the north-east from the MPA nuclear facilities. Estimated external doses for residents living in the highest contaminated settlements that were evacuated within the first 10 days following the accident were 650–1,300 mGy, while they were 50–270 mGy in settlements at a distance of 35–55 km from the site of release, which were evacuated within the first 250 days following the accident. In less contaminated settlements, which were not evacuated, estimated external dose was below 50 mGy cumulated over lifetime.

At the Lake Karachay, another smaller incident occurred with release of mixed fission products in April 1967 that formed the Karachay Trace over an area of 1,800 km^2^ (Peremyslova et al. 2004). About 0.2 PBq of activity, mainly ^90^Sr and ^137^Cs, was released as a result of downwind transfer of shoreline and bottom sediments from the partially dried-out lake.

In the current study, the absorbed dose in tooth enamel from citizens of Ozyorsk who were never employed by MPA was measured with EPR. The aim of the study was to assess the external exposure of the citizens during residence at Ozyorsk, due to natural background radiation and due to radioactive noble gases routinely released by MPA. Additionally, emphasis was placed on the identification of further time periods with external exposures others than those known for routine atmospheric MPA releases.

## Materials and methods

### Physical background

In EPR dosimetry with tooth enamel, the absorbed dose is evaluated from the content of carbon dioxide radicals that were generated by ionising radiation from carbonate impurities in hydroxyl apatite, the main component of tooth enamel (Fattibene and Callens 2010). The energy required for the formation of such a radical is about 5 eV (UVC: 250 nm), which is much lower than the 34 eV required for the ionisation of air (Wieser 2012). Furthermore, due to the high calcium content of hydroxyl apatite, photons with low energy are absorbed more significantly than in air. Hence, EPR dosimetry with tooth enamel can detect exposures from photons with low energy more effectively than measurements with ionisation chambers. This allows the detection of external exposure from natural background radiation, ^41^Ar from routine radioactive noble gas releases from nuclear facilities, and ^133^Xe, the main component of fission product noble gases, from accidental releases. During decay, ^133^Xe emits beta, gamma, and X-ray radiation with a mean energy of 100, 81, and 2 keV, respectively (ICRP 2008). The absorbed dose in tooth enamel from beta and gamma exposure is about 10 times larger than air kerma from gamma exposure (Ulanovsky et al. 2005). The contribution of the X-rays to absorbed dose in enamel has not yet been evaluated. In contrast, ^41^Ar emits gamma and beta radiation with a mean energy of 1,300 and 460 keV, respectively. The absorbed dose in tooth enamel, from gamma and beta exposure, is about 40 % larger than air kerma from gamma exposure.

### Samples

The present study included molars from the citizens of Ozyorsk who were never employed at MPA and who are not members of the Techa River Cohort. Two hundred and twenty-nine teeth were collected in the dental clinic of Ozyorsk from two hundred citizens born between 1922 and 1971. Twenty-one of these citizens donated two teeth, and four citizens donated three teeth. From 1995 to 2006, the teeth were extracted due to medical indications, and patient’s agreement was obtained to donate them for EPR analysis. For each tooth donor, a record was compiled containing information on date of tooth extraction, tooth position, the donor’s name, date of birth, and year of arrival at Ozyorsk. Additional information on places of birth and emigration, and dose-relevant individual activities were obtained from the database of the Mayak Children’s Cohort (Koshurnikova et al. 2002). Here, the citizen’s last place of residence before moving to Ozyorsk was called the place of emigration, and the year when the individual moved to Ozyorsk was called the year of arrival. About half of the citizens moved to Ozyorsk before 1960, and about 10 % of the citizens were born in Ozyorsk.

### EPR measurements

All EPR measurements were taken in the framework of three projects funded by the European Commission: (a) INCO-COPERNICUS ‘Dose Reconstruction for Workers of Mayak and Techa Riverside Residents’, (b) FP5 ‘EPR Dose Reconstruction with Teeth: In situ Measurements, Determination of Organ Doses, and Application at Mayak (DOSREC)’, and (c) FP6 ‘Southern Urals Radiation Risk Research (SOUL)’. They were performed at the Helmholtz Zentrum München, Germany, and at the Istituto Superiore di Sanità, Italy, from 1998 to 2009. The enamel samples were separated chemically from the teeth and spectrum deconvolution was applied for the evaluation of the radiation-induced signal intensity from the tooth enamel EPR spectra. Further details on the applied procedures and on EPR dosimetry with tooth enamel can be found elsewhere (Onori et al. 2000; Wieser et al. 2001; Fattibene and Callens 2010). Evaluation in terms of absorbed dose in enamel was done based on a calibration curve that was established by the irradiation of enamel reference samples under electronic equilibrium conditions with a ^60^Co source. With improvements in measurement technique, the standard error of dose evaluation decreased from 90 to 40 mGy for measurements in 1998 and after 2004, respectively. This resulted in decreasing critical dose levels (CDL) from 150 to 60 mGy. Samples with exposure below the CDL cannot be distinguished from unexposed samples (Wieser et al. 2008; Fattibene et al. 2011). Therefore, in the present study, all measurement results below the CDL were set to the half-value of the CDL, which was considered as the most probable dose.

### Analysis of measurements

The analysis of the dose measured for molars (tooth positions 4–8) was based on person age and the corresponding stage of tooth development at the time of exposure (Schröder 2000, Table 1). The table indicates that variability of average age, at the various stages of tooth development for teeth from upper and lower jaws, was less than ±1 year for most tooth positions. For the other positions, the maximum and minimum values are given in parentheses in Table 1. Tooth completion was defined by the complete eruption of the tooth with the end of tooth root development. With tooth completion, tooth enamel can detect exposures with the widest energy spectrum, including external beta exposure, and exposures from soft photons that result typically from accidentally released radioactive noble gas isotopes but not from the routinely released isotopes. In contrast, with crown completion, where the enamel is still covered by gums, the enamel is less sensitive to soft photon and beta exposures. The crown is completed on average 6 years before complete eruption of the tooth (tooth completion). Here, the time period between crown completion and the year of EPR measurement was called crown age and was used to calculate the external annual gamma dose from natural background exposure with minor contributions from soft photons. The time period between tooth completion and the year of EPR measurement will be called in the following as tooth age. The year of tooth completion was used to identify the periods of exposures.

**Table 1:** Average person age at different stages of tooth development

Tooth position	Beginning of mineralisation	Crown completion	Tooth completion
1	0	4	9
2	1	5 (3–6)	10
3	1	5	12
4	2	6	12 (11–14)
5	3	7	13
6	0	3	9 (8–11)
7	3	7	14 (11–16)
8	9	13	20

All numbers are in years; values were extracted from Schröder (2000). Maximum and minimum values are given in parentheses if variability was larger ±1 year

In the phase of tooth mineralisation, radionuclides like ^90^Sr can be incorporated in tooth enamel with the largest uptake occurring at the beginning of mineralisation (Shishkina et al. 2011). In the present study, teeth with the beginning of mineralisation after 1950 were checked non-destructively for their ^90^Sr content by beta spectrometry (Semioshkina et al. 2011). The detection limit was 0.6 Bq/g. This allowed for the assessment of an internal dose contribution in enamel of at least 300 mGy, accumulated within 50 years after uptake.

## Results and discussion

### Distribution of measured doses

**Fig. 1:**
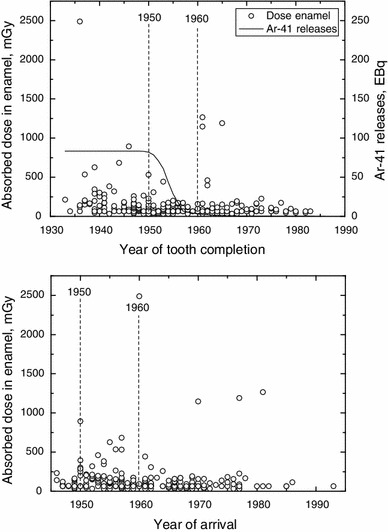
Absorbed dose in enamel of 229 teeth from citizens of Ozyorsk versus the year of tooth completion (*top*) and arrival in Ozyorsk (*down*). Also shown are the cumulated routine ^41^Ar releases of MPA following the year of tooth completion (*top*, *solid line*). The *dashed lines* indicate end and beginning of the early and late operation period of MPA facilities in 1950 and 1960, respectively. EBq corresponds to 1 × 10^18^ Bq

The absorbed dose in tooth enamel, measured by EPR in 229 teeth of citizens of Ozyorsk, is shown in Fig. 1 in dependence on the year of tooth completion and the citizen’s arrival at Ozyorsk. The absorbed dose was less than 200 mGy in 88 % of the measured teeth and below 100 mGy in 60 %. Out of the remaining teeth, a large dose of more than 300 mGy was measured in seventeen teeth (7 % of all) including nine teeth (4 % of all) with doses in excess of 500 mGy. The largest doses were measured in teeth which were completed before 1950 as well as in those completed after 1960, in the early and late period of MPA facility operation, respectively. The main routine releases of radioactive noble gases from MPA occurred with 95 % of the total activity between these operation periods, from 1950–60, while 2 and 3 % were released before 1950 and after 1960, respectively. The main component of the routinely released radioactive noble gas isotopes was ^41^Ar; its cumulated release following the year of tooth completion is also shown in Fig. 1. Because the releases started in 1948, the cumulated release following tooth completion is maximal and constant for all teeth which were completed before 1948.

### Identification of circumstances and periods of external exposure

As already indicated, the aim of the present study was to assess the exposures of the citizens to natural background radiation and routine noble gas releases from MPA, during residence at Ozyorsk. Therefore, before the analysis, citizens had to be identified with indication of having received possibly significant exposures during other periods than those known for main routine atmospheric releases between 1950 and 1960. This was done based on the measured dose level, period of tooth formation, year of arrival of the person at Ozyorsk, place of emigration, and dose-relevant individual activities. Here, the period of tooth formation covers the calendar years from the beginning of tooth mineralisation to the end of tooth eruption (tooth completion, see Table 1).

Based on the assessments of absorbed dose in molars from natural background radiation made by other authors, the annual absorbed dose was estimated to be typically about 1 mGy for populations from the former Soviet Union (Ivannikov et al. 1997, 2006, Takada et al. 1997). Similar annually absorbed doses can be expected also for the populations in the Urals. The crowns of the molars from this study were all younger than 80 years, and with a standard error (SE) of 40 mGy for EPR measurements, the anticipated dose to be measured in enamel from natural background exposure would be less than 200 mGy, with 99 % (3 SEs) confidence. Due to the expected relatively low-dose contribution from routine noble gas releases (Glagolenko et al. 2008c), the upper dose level of 200 mGy was considered to lie above, or for the oldest teeth close to, the expected combined dose contributions in enamel from natural background radiation and routine noble gas releases. Therefore, as a starting point, all the citizens with an absorbed dose in enamel of more than 200 mGy were evaluated for the indications of possible additional exposures outside of the period of routine releases. In 28 teeth from 26 individuals, the absorbed dose in enamel was found to be above 200 mGy. After the evaluation of year of tooth completion, arrival at Ozyorsk, place of emigration, and dose-relevant individual activities, indications of additional exposures other than natural background radiation and routine noble gas releases were found possible for 24 teeth of 22 citizens, corresponding to about 10 % of all citizens involved in this study. For the remaining four teeth from four individuals, with absorbed doses between 205 and 232 mGy, no indication of possible exposures outside of the period of routine releases was obvious.

Information about these 22 citizens with possible additional exposure is provided in Table 2. The information includes person ID, years of birth and arrival at Ozyorsk, period of tooth formation, absorbed dose in enamel, distance to MPA discharges from last residence before arrival at Ozyorsk, and comments on places of birth and emigration (last residence), and on individual activities. Additional exposure was found possible for five individuals (IDs 1871, 1885, 1975, 1728, and 2033) because they had moved to Ozyorsk from contaminated areas of the EURT, for three individuals (IDs 1735, 1083, and 993) due to participation in liquidation of the Kyshtym accident, and for further three individuals (IDs 479, 354, and 1802) because of their work as external service personnel at the MPA premises.

**Table 2:** Ozyorsk citizens with indications of possible exposures other than from background radiation and routine noble gas releases

ID	Birth	Arrival Ozyorsk	Tooth formation	Dose (mGy)^a^	Distance MPA (km)^b^	Comment
*Emigrants from the EURT area*						
1871	1924	1960	1926–1936	2,490	50	Birth and emigration: Bagaryak, Chelyabinsk region
1885	1935	1957	1935–1944	683	30	Birth and emigration: Kazhakulj, Chelyabinsk region
1975	1952	1977	1955–1965	1,190	40	Birth: Kasli, Chelyabinsk region Emigration: South Konevo, Chelyabinsk region ^90^Sr in enamel: 2.1 Bq/g in 2009, internal dose: 800 mGy
1728	1948	1970	1951–1961	1,150	40	Birth and emigration: Golubinka, Chelyabinsk region ^90^Sr in enamel: below detection limit
2033	1959	1977	1962–1973	226	40	Birth and emigration: Tataskaya Karabolka, Chelyabinsk region ^90^Sr in enamel: below detection limit
*Liquidators of the Kyshtym accident in 1957*						
1735	1936	1957	1939–1950	532	20	Birth: Chelyabinsk Emigration: Kyshtym, Chelyabinsk region Medical radiation treatment
1083	1928	1954	1931–1942	383	30	Birth: Sverdlovsk region Emigration: Upper Ufaley, Chelyabinsk region
993	1931	1952	1933–1943	318	1,500	Birth and emigration: Smolensk region 1952–59 engine driver at MPA site
*External service personnel at MPA*						
479	1927	1955	1929–1939	626	20	Birth and emigration: Kasli, Chelyabinsk region Frequent visits of MPA
354	1926	1950	1929–1939	288	1,000	Birth and emigration: Tataria Since 1950 security stuff at MPA auxiliary departments
1802	1936	1956	1939–1949	264	500	Birth and emigration: Kuzyakovo, Bashkiriya Since 1956 cleaning of working clothes at MPA
*Citizens immigrated since 1950 from MPA neighbourhood*						
1013	1926	1950	1935–1946	893	100	Birth: Gorky region Emigration: Chelyabinsk Occupation: Medical organisation Ozyorsk
1914	1928	1950	1930–1940	278^c^	20	Birth: Panarevo, Kurgan region Emigration: Kyshtym, Chelyabinsk region Occupation: Medical organisation Ozyorsk
*Citizens born in Ozyorsk with tooth completion after 1953*						
1861	1953	1953	1953–1962	460	15	Birth: Ozyorsk ^90^Sr in enamel: below detection limit
2013	1950	1950	1952–1962	394	15	Birth: Ozyorsk ^90^Sr in enamel: below detection limit
*Citizens immigrated from far distant places after 1953*						
1930	1925	1956	1927–1937	535	1,700	Birth and emigration: Moscow Occupation: Actor
1865	1941	1961	1943–1953	442	1,400	Birth and emigration: Novgorod Occupation: Trading
1347	1930	1954	1930–1939	344	1,700	Birth and emigration: Moscow Occupation: Radio-chemist at FIB-1
494	1938	1962	1941–1951	308	500	Birth and emigration: Permj region Occupation: Medical organisation Ozyorsk
2000	1938	1964	1938–1947	258	1,500	Birth and emigration: Tejkovo, Ivanovskaya oblastj Occupation: Medical organisation Ozyorsk
1107	1930	1957	1933–1944	227	1,800	Birth and emigration: Vologda region Occupation: Medical organisation Ozyorsk
762	1952	1981	1952–1961	1,270	500	Birth and emigration: Bashkiria 1971–1975 resident of Tajikistan Occupation: X-ray service at research centre ^90^Sr in enamel: below detection limit

*EURT* East Urals Radioactive Trace

^a^Absorbed dose in enamel

^b^Distance of place of emigration to site of MPA discharges

^c^Mean of 3 teeth: 302, 279, and 252 mGy

Before start of main routine noble gas releases in 1951, citizens could have been exposed during the early MPA operation period in 1948–1950. The possible releases from this period could have been affected the citizens during residence at Ozyorsk, or before moving to Ozyorsk, if previous residence at that time was in the vicinity to the MPA facilities, up to about 100 km. Exposure before moving to Ozyorsk was found possible for two individuals (IDs 1013 and 1914), with teeth completed before 1948, who arrived from the MPA vicinity, in 1950. Note that releases from the early period of MPA operation could affect absorbed dose in enamel only if teeth were completed latest in 1950 and donors lived in Ozyorsk or MPA vicinity in 1948–1950.

After 1953, the year of the peak of routine noble gas releases, excess doses in enamel could result from potential releases in the late period of MPA operation. The enamel of the citizens could have been affected during residence at Ozyorsk, or before moving to Ozyorsk, if previous residence was in the MPA vicinity. Absorbed dose contributions in enamel during residence at Ozyorsk would be dominated by exposure from these releases, if the citizens were born in Ozyorsk and the corresponding teeth were completed after 1953, or if the tooth donors arrived with completed teeth at Ozyorsk after 1953, from far distant places with no anthropogenic exposures. During residence at Ozyorsk, dominance of doses from potential releases from the late MPA operation period was found possible for two individuals (IDs 1861 and 2013) who were born in Ozyorsk, with teeth completed in 1962, and further seven individuals (IDs 1930, 1865, 1337, 494, 2000, 1107, and 762) who arrived from far distant places, after 1953.

### Absorbed dose from natural background exposure

**Fig. 2:**
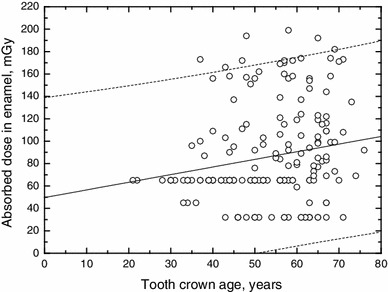
Absorbed doses in enamel of 182 teeth from 161 citizens who entered Ozyorsk after 1953, as a function of crown age; individuals were exposed essentially to natural background radiation and routine noble gas releases of MPA. Also shown are the linear least squares fitting curve (*solid line*) of the data and the 95 % prediction interval (*dashed lines*)

There was no evidence of significant additional exposures outside of the period of routine noble gas releases for the remaining 205 teeth from 178 individuals. Out of these, 182 teeth were donated by 161 citizens arriving at Ozyorsk after 1953, or with birth in Ozyorsk and teeth completed after 1953, the year of maximal routine releases of radioactive noble gases. The absorbed doses in tooth enamel of these teeth are shown in Fig. 2, as a function of the age of the tooth crown at the time of EPR measurement. Because the standard error of an individual EPR measurement was 40 mGy, EPR dosimetry cannot provide accurately the relatively low dose from natural background radiation for an individual, but can provide an estimate for the average dose of a larger group of individuals. The unweighted linear regression analysis for the 182 EPR measurements resulted in an enamel dose-to-crown age response curve with an intercept (bias) of (50 ± 15) mGy and a slope of (0.7 ± 0.3) mGy/y if the crown age is given in years. The residual standard deviation between measured and fitted doses was 43 mGy, which was calculated as root mean squares of the deviations between measured and the fitted dose at the corresponding crown age. The annually absorbed dose in enamel of (0.7 ± 0.3) mGy corresponds to a dose rate of (80 ± 30) nGy/h, which is close to the dose rate of 70 nGy/h from natural background radiation in Ozyorsk (Glagolenko et al. 2008c).

Since 1959, the gamma dose rate from gaseous MPA releases was measured with an automatic monitoring system in Ozyorsk (Glagolenko et al. 2008c). These measurements were taken at the Meteostation, north-east of the city at the shore of Lake Irtyash, in 1959–1992, and at the MPA central physical laboratory, in the West of the city, in 1960–1968. During the period of simultaneous measurements, doses at the central physical laboratory were on average approximately half of those measured at the Meteostation. In the time period 1959–1992, at the Meteostation, the accumulated gamma dose was 3.5 mGy with an average annual dose of 0.25 mGy until 1967, while the annual dose was less than 0.05 mGy afterwards. Thus, these additional dose rates from gaseous MPA releases are within the uncertainties of the annual dose in teeth evaluated by EPR.

The bias of the EPR-evaluated annual dose in these teeth of 50 ± 15 mGy is close to 33 mGy, which was estimated as internal dose to 50-year-old enamel, from radionuclides (mainly ^238^U, ^40^K, ^90^Sr) found in teeth of the general European population (Wieser 2012). Radionuclides incorporated by an individual will contribute to the annual dose to that sample. However, because of the individual variability in the uptake of radionuclides, and the pronounced time dependence of ^90^Sr present in the environment due to global fallout, MPA releases and accidents, radionuclides are generally not homogeneously incorporated in all teeth of a population, and in particular in the tooth crowns which were completed in the same year from different individuals. Furthermore, EPR measurements were taken within a period of 12 years (1998–2009), and consequently, tooth crowns of the same age (time period between crown completion and EPR measurement) as given in Fig. 2 were not completed in the same year but crown completion as well as start of tooth mineralisation occurred within a period of 12 years with variable conditions of radionuclide uptake. The difference in the means of the internally absorbed doses in enamel of different age from different individuals results mainly due to differences in the radionuclide content, with minor influence due to differences in age of the enamel. Hence, on average, over the teeth from a population, the average internally absorbed dose due to incorporated radionuclides contributes to the bias of the enamel dose-to-crown age regression curve, with minor influence of the individual annually absorbed dose, due to incorporated radionuclides, to the slope of the curve.

A further contribution to the bias can result from accidents after 1953 and possible releases from the late MPA operation period. These releases would affect the absorbed dose in enamel of all teeth completed at the time of exposure, independent on the crown age and hence contribute to the dose bias. From the measurements presented in Fig. 2, a sharp increase in the measured absorbed doses is obvious for crowns older than about 35 years, which contributes to the bias of the regression curve. For crowns with age between 35 and 50 years, tooth mineralisation started between 1951 and 1966, the period with ^90^Sr releases from MPA in 1950–1956, the Kyshtym accident in 1957, and the Lake Karachay incident in 1967, and any uptake of ^90^Sr in enamel might have contributed to the internal dose to these teeth. For crowns older than about 50 years, teeth were completed latest in 1956 and, accordingly, all of them accumulated external doses from the Kyshtym accident as well as from potential gaseous releases from the late MPA operation period after 1960. Note that tooth mineralisation starts on average 4 years before crown completion and the tooth will be completed on average 6 years later than the crown (see Table 1). Therefore, teeth with beginning of mineralisation in 1950–1956, the period of main ^90^Sr releases of MPA, were completed in 1960–1966 and can include internal dose contributions from ^90^Sr as well as external dose contributions from potential gaseous releases of the late MPA operation period.

To conclude, after 1953, the annually absorbed dose in tooth enamel during residence at Ozyorsk can be considered to result mainly from natural background radiation, with only minor contributions from routine noble gas releases of MPA. The absorbed dose bias of 50 ± 15 mGy in enamel of these teeth can include internal dose contributions due to incorporated naturally occurring radionuclides, and due to ^90^Sr from global fallout, MPA accidents and routine releases, as well as external dose contributions due to accidents at MPA and possible releases of the late MPA operation period since 1957.

### Dose contribution from routine noble gas releases

**Fig. 3:**
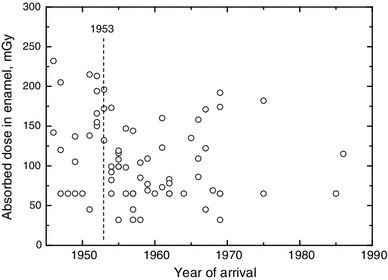
Absorbed doses in enamel of teeth completed before 1951 as a function of arrival year at Ozyorsk. The *dashed line* indicates the year of maximal routine releases of MPA

The absorbed dose in enamel of 78 teeth from 70 individuals, which were completed before 1951, is shown in Fig. 3 as a function of arrival year. Note that in 1951, the major routine noble gas releases from the MPA started. The absorbed dose in 23 teeth from 21 individuals of those citizens arriving at Ozyorsk until 1953 was higher than expected from background radiation (Table 3), on average by 36 ± 29 mGy. In contrast, good agreement between the measured dose in enamel and the expected dose from background radiation was found in 55 teeth from 49 individuals of those citizens arriving after 1953 (i.e. expected from the regression curve shown in Fig. 2). The outdoor external doses resulting from model calculations of the routine noble gas releases at MPA were estimated for the locations of the two measurement stations in Ozyorsk, the MPA central physical laboratory (in the West of the city) and the Meteostation (in the north-east of the city) (Glagolenko et al. 2008c). In the time period 1949–1989, the estimated cumulated external gamma dose was 13 and 19 mGy at the central physical laboratory and the Meteostation, respectively. These gamma doses resulted, as already mentioned, predominantly from ^41^Ar. An additional beta dose contribution from ^41^Ar of about 40 % would be detected additionally in tooth enamel (see above) resulting in total doses in tooth enamel of 18 and 27 mGy. These estimated doses in enamel are consistent with the measured excess dose of 36 ± 29 mGy in teeth of citizens who moved to Ozyorsk until 1953. Maximal dose contribution from routine noble gas releases is expected in teeth of the citizens arriving until 1950, because they were exposed to the total releases cumulated after 1950. Cumulated releases since arrival at Ozyorsk, and resulting absorbed doses, will decrease for the citizens arriving later on. In 1951–1952, about 20 % of the total activity of noble gases was released, and hence, the absorbed dose in teeth is expected to be about 20 % below maximum for the citizens who arrived only in 1953. Until 1950, 10 citizens (12 teeth) moved to Ozyorsk with measured average excess dose above background of (14 ± 30) mGy (Table 3). A larger average excess dose of (60 ± 30) mGy was measured for 11 citizens (11 teeth) who arrived at Ozyorsk later on in 1951–1953. Within measurement uncertainty estimated excess doses for the two subgroups of citizens are in agreement, but there seems to be the tendency that the excess dose is larger for the citizens arriving in 1951–1953. This can be caused due to differences in exposure activity at the individual places of living in Ozyorsk (which are unknown) and/or that part of the citizens arriving in 1951–1953 came from places in MPA vicinity (which had not yet been evaluated) with possible additional exposures before arrival.

**Table 3:** Measured average doses in enamel for 78 teeth completed before 1951, from citizens of Ozyorsk, and calculated background dose

Year of arrival	Number of teeth	Crown age (years)	Measured average dose (mGy)	Calculated average background dose (mGy)^a^	Excess measured dose (mGy)
1946–1950	12	67 ± 6	111 ± 17	97 ± 25	14 ± 30
1951–1953	11	73 ± 6	161 ± 14	101 ± 27	60 ± 30
1946–1953	23	70 ± 7	135 ± 12	99 ± 26	36 ± 29
1954–1986	55	65 ± 5	93 ± 6	96 ± 25	−3 ± 26

^a^Including accumulated annual dose and dose bias, calculated from crown age and parameters of regression curve of measurements shown in Fig. 2

Additional dose contributions during residence at Ozyorsk from releases that might have occurred in the early period of MPA operation before 1951 should be observable in teeth of citizens who arrived until 1950, but not later. Because the measured average excess dose (14 ± 30 mGy) above background, in teeth from the subgroup of citizens arriving until 1950, was very close to the expected doses from routine noble gas releases, there is currently no evidence of dose contributions during residence at Ozyorsk from releases before 1951. Note that from the 200 citizens included in this study, only ten moved to Ozyorsk until 1950 and could have been affected from the early releases during their residence at Ozyorsk. More measurements of teeth from this subgroup would be needed in order to confirm or withdraw the preliminary conclusion that during residence at Ozyorsk, citizens were not affected by potential MPA releases before 1951.

To conclude, until 1953, the exposure of teeth during residence at Ozyorsk can be considered to result mainly from background radiation, as is the case for the exposure of teeth of citizens arriving after 1953, but with additional contributions from routine noble gas releases. The currently available EPR measurements showed no indication that during residence at Ozyorsk, citizens were affected by potential MPA releases before 1951.

### Teeth with exposures at different time periods

The teeth with indications of additional exposure listed in Table 2 include six teeth with tooth completion in the late period of MPA operation after 1960 (IDs 1975, 1728, 2033, 1861, 2013, and 762). Mineralisation of these teeth began in 1951–1962, and ^90^Sr could have been incorporated in enamel from liquid MPA releases after 1950 or from the contamination from the Kyshtym accident in 1957. Interestingly, beta spectrometry measurements detected ^90^Sr contamination only in one of these teeth. This tooth was donated from a person who moved to Ozyorsk from a place in the EURT in 1977 (see ID 1975 in Table 2). The tooth crown was formed in 1955–1959 at the time of contamination from the Kyshtym accident. In 2009, the measured ^90^Sr/^90^Y activity in tooth enamel was 2.1 ± 1.1 Bq/g. The estimated cumulated internal dose was 800 ± 400 mGy, 50 years after uptake of ^90^Sr in 1957, calculated with a dose rate coefficient of 0.056 nGy/g/s, assuming only radioactive decay without biological removal of ^90^Sr from enamel (Tikunov et al. 2006), which is close to the measured total absorbed dose in enamel of 1,190 mGy. ^90^Sr content below detection limit was found for one tooth (ID 2033) with absorbed dose in enamel of 226 mGy. There was no evidence of ^90^Sr contamination of the other teeth, which thus must have been exposed externally after crown or tooth completion. The highest doses of about 1,200 mGy were measured in two teeth, both completed in 1961. The corresponding tooth donors (IDs 1728 and 762) entered Ozyorsk in 1970 and 1981, respectively, and it is considered likely that exposure of the teeth occurred before moving to Ozyorsk, between 1955 (crown completion) and 1961 (tooth completion) and the individual’s arrival at Ozyorsk. Note that such huge absorbed doses in enamel, which are more than an order of magnitude larger than the doses measured from the routine noble gas releases, could be due to exposure with low-energy photons from noble gas isotopes released in the course of accidents. One of the citizens lived at a place of the EURT before arriving in Ozyorsk, which makes it possible that between tooth completion in 1961 and arrival to Ozyorsk in 1970, the tooth was exposed by low-energy photons from potential accidental noble gas releases from the late MPA operation period. The other citizen emigrated from a far distant place only in 1981 and was engaged before with the service of X-ray equipment (see Table 2). It could not be clarified whether the exposure occurred during his professional activity or must have been occurred afterwards in Ozyorsk. Exposure during residence at Ozyorsk from potential accidental releases was found possible for the remaining two citizens (IDs 1861 and 2013), with teeth completed in 1962 during the late period of MPA operation. These citizens were born in Ozyorsk, and the absorbed doses in enamel of about 400 mGy must result from exposures after tooth completion in 1962.

Exposure from accidental releases during residence at Ozyorsk was found possible for further seven citizens, six of them (IDs 1930, 1865, 1347, 494, 2000, and 1107) came to Ozyorsk from far distant places in 1954–1964, while one (ID 1802) belonged to the MPA external service personnel and moved to Ozyorsk in 1956, with absorbed doses in enamel between 227 and 535 mGy. To summarise, in total nine individuals were exposed after the main MPA routine releases, four of them arrived at Ozyorsk in 1954–1957 (IDs 1802, 1930, 1347, and 1107), and five arrived in 1961–1964, (IDs 1865, 494, and 2000) or at least their teeth were completed (IDs 1861 and 2013) in 1962, with average absorbed doses in enamel of (343 ± 137) mGy and (372 ± 87) mGy, respectively. The absorbed doses in enamel from these two subgroups are very close to each other and can be the result of the same event that could have happened earliest in the year in which the last of these citizens arrived at Ozyorsk, i.e. in 1964. Note that the measured absorbed dose in enamel (264 mGy) for the person from the MPA external service personnel (ID 1802) was well within the range of absorbed doses in enamel from the other persons who arrived at Ozyorsk since 1954, and there was no evidence of an additional dose contribution from the work at MPA premises.

The absorbed dose in enamel of another person from the external service personnel (288 mGy, ID 354), who arrived earlier in 1950, and that in enamel of a person involved in liquidation of the Kyshtym accident (318 mGy, ID 993), who arrived in 1952, were slightly higher, which could be explained by the contribution of routine noble gas releases in 1950–1952. Hence, for this individual, there was no evidence of a significant additional dose contribution during liquidation.

To conclude, excess absorbed doses were measured in enamel of 11 individuals, due to exposures after the peak of routine noble gas releases from MPA in 1953. These exposures must have been occurred during residence of these individuals in Ozyorsk resulting in an average absorbed dose in enamel of (349 ± 97) mGy, with no obvious dependence of the absorbed dose on the year of the individual’s arrival at Ozyorsk. These exposures could be explained by a single event that must have happened in 1964 or later. The average absorbed dose from this event during the late MPA operation period is approximately an order of magnitude larger than the measured absorbed dose (36 ± 29 mGy) due to the routine noble gas releases and could be due to exposure with low-energy photons from noble gas isotopes.

In teeth from seven citizens (IDs 1871, 1885, 1735, 1083, 479, 1013, and 1914) whose teeth were completed until 1950 and who moved in from the MPA vicinity, absorbed doses from 278 to 2,490 mGy were measured. Five citizens moved to Ozyorsk in 1954–1960 and two in 1950. These individuals could have received exposures before arrival from the releases of the early MPA operation period, as well as exposures during residence at Ozyorsk from the releases of the late MPA operation period and routine noble gas releases. In the enamel from four citizens (IDs 1871, 1885, 479, and 1013), the absorbed dose (626–2,490 mGy) is larger than the doses measured in enamel from the citizens who were exposed by routine noble gas releases or in the late MPA operation period (see above). These teeth were completed latest in 1944, and the first of these citizens entered Ozyorsk in 1950. Hence, the possible exposure period of the enamel started with tooth completion before the individuals moved to Ozyorsk and covers—for all four citizens—the period of early MPA operation from 1948 to 1950, before the start of the major routine noble gas releases of MPA. The largest dose of about 2,500 mGy was measured in the tooth from a citizen (ID 1871) who emigrated in 1960 from Bagaryak located at a distance of 50 km north-east of the MPA facilities. This place is located within the area of the EURT and was contaminated by releases from the Kyshtym accident in 1957. The population of Bagaryak was not evacuated after the accident, and maximal external dose to the citizens was estimated to about 50 mGy (Peremyslova et al. 2004). Thus, it is unlikely that the high dose measured in the tooth of this citizen was caused by the contamination in 1957. The common possible exposure period for the teeth from these four citizens, before moving to Ozyorsk, is the early MPA operation period from 1948 to 1950, and the absorbed doses in enamel could result from exposure by accidental noble gas releases (mainly ^133^Xe) that might have happened in the early operation period of the first MPA reactor until 1950.

## Conclusions

The aim of the present study was to assess the external exposure of citizens during residence at Ozyorsk, due to natural background radiation and due to routinely released radioactive noble gases by MPA. Further emphasis was placed on the identification of further time periods with external exposures, in addition to those for routine atmospheric releases. In the current investigation, the absorbed dose in tooth enamel was analysed from EPR measurements of 229 teeth from citizens of Ozyorsk. The citizens were not employed at the MPA nuclear facilities and were born between 1922 and 1971. About half of the teeth originated from citizens who arrived at Ozyorsk before 1960 and about 10 % of the citizens were born in Ozyorsk. The absorbed dose was less than 200 mGy in 88 % of all teeth and more than 500 mGy in 4 %. Before the analysis, those citizens had been identified who indicated possible exposures outside of the period of routine noble gas releases. This was done on the basis of the measured dose level, period of tooth formation, arrival of the individual at Ozyorsk, and the place of former residence.

In 90 % of the citizens involved in the present study, no indication of exposures outside of the period of the MPA routine noble gas releases was found. In teeth from those citizens who arrived at Ozyorsk after 1953, the annually absorbed dose in enamel was (0.7 ± 0.3) mGy, corresponding to a dose rate of (80 ± 30) nGy/h, which is close to the dose rate of 70 nGy/h from natural background radiation in Ozyorsk. After 1953, the exposure of teeth for individuals living in Ozyorsk can be considered to include only minor contributions from routine noble gas releases. In teeth from citizens who moved earlier to Ozyorsk, i.e. in 1953 or before, the average excess absorbed dose in enamel above natural background was 36 ± 29 mGy, which is close to the expected absorbed dose in enamel from routine noble gas releases of the MPA, which peaked in 1953. This is consistent with the dose in enamel from the residents of Ozyorsk due to routine noble gas releases, including the beta dose contribution, which was estimated to be 18–27 mGy in model calculations.

Indication of exposure during residence at Ozyorsk from accidental noble gas releases from the late MPA operation period was found in citizens who were born in Ozyorsk and in emigrants from far distant places. The absorbed doses in enamel of these citizens were 227–535 mGy with an expected exposure in 1964 or later. Additional EPR measurements of teeth from citizens born before 1960 in Ozyorsk will be necessary for further clarification of these accidental noble gas releases. The majority of citizens from this study with indications of additional exposures could have been exposed during residence at Ozyorsk from accidental noble gas releases after 1960. There was no evidence of additional doses for those citizens who had been involved in liquidation of the Kyshtym accident and for the external MPA service personnel, due to the liquidation work or work at the MPA premises.

Out of the teeth completed until 1944 before the early period of MPA operation, absorbed doses of 626–2,490 mGy were measured in teeth from four citizens who emigrated from the MPA vicinity. For these individuals, the exposure period can be expected in the early period of MPA operation from 1948 to 1950 before they arrived at Ozyorsk and before the major routine noble gas releases of MPA started. The largest dose of 2,490 mGy was measured in the tooth from an individual who emigrated from a place in the EURT located at a distance of 50 km north-east of the MPA facilities. It is unlikely that the high dose in the tooth of this citizen was caused by the contamination from the Kyshtym accident in 1957; rather, it is more likely that it was caused by earlier accidental noble gas releases (mainly ^133^Xe) that might have happened in the early operation period of the first MPA reactor until 1950. Further EPR measurements of teeth from citizens who emigrated from the MPA vicinity before 1960 will be necessary for further clarification of the exposure period. There is currently no further evidence of an additional dose contribution during residence at Ozyorsk from accidental noble gas releases from the early period of MPA operation until 1950.

The citizens involved in this study were randomly selected and do not represent the population structure during the early and late periods of MPA operation. Therefore, the current measurements cannot be considered to reproduce the actual radiation situation during earlier MPA operation periods. Rather, they can give only indications for possible accidental gaseous releases from the MPA, while they cannot provide precise estimates of the released activities. The main health risk to the population from accidental gaseous releases results from the accompanying radioactive iodine isotopes. The ^131^I isotope, with a physical half-life of about 8 days, is decaying to the long-lived ^129^I isotope, which can be detected retrospectively by accelerator mass spectrometry (AMS) in environmental samples (e.g. Reithmeier et al. 2010). The detection of ^129^I in the rings of trees in Ozyorsk and the MPA vicinity could be a reliable method for a time-resolved assessment of any accidental gaseous releases that might have happened during the operation of the MPA facilities.
